# Dogs as victims of their own worms: Serodiagnosis of canine alveolar echinococcosis

**DOI:** 10.1186/s13071-017-2369-0

**Published:** 2017-09-16

**Authors:** C.F. Frey, N. Marreros, S. Renneker, L. Schmidt, H. Sager, B. Hentrich, S. Milesi, B. Gottstein

**Affiliations:** 1 Department of Infectious Diseases and Pathobiology, Vetsuisse Faculty, Institute of Parasitology, University of Bern, Länggassstrasse 122, CH-3001 Bern, Switzerland; 2 Canadian Food Inspection Agency, Centre for Food-borne and Animal Parasitology, 116 Veterinary Road, Saskatoon, S7N 2R3 Canada; 3 EUROIMMUN AG, Seekamp 31, 23560 Luebeck, Germany; 4 Elanco Animal Health, Schwarzwaldallee 215, CH-4058 Basel, Switzerland

**Keywords:** *Echinococcus multilocularis*, Alveolar echinococcosis, Dog, ELISA, Western blot, RecEm95

## Abstract

**Background:**

Besides acting as definitive hosts for *Echinococcus multilocularis*, dogs can become infected by the larval form of this parasite and thereby develop life-threatening alveolar echinococcosis (AE). Although AE is a zoonotic disease, most therapeutic and diagnostic approaches have been developed for human patients. In dogs, AE is typically diagnosed in the advanced stage of the disease when the parasitic mass has already caused abdominal distension. At that stage, complete resection of the parasitic mass is often impossible, leaving a guarded prognosis for the affected dogs. For humans, sensitive and specific diagnostic protocols relying on serology have been validated and are now widely used. In contrast, sensitive and specific laboratory diagnostic tools that would enable early diagnosis of canine AE are still lacking. The aim of the current study was to establish a serological protocol specifically adapted to dogs.

**Methods:**

We tested several native and recombinant antigens (EmVF, Em2, recEm95, recEm18) in in-house ELISA, an in-house Western blot (WB), as well as a commercially available WB developed for serodiagnosing human AE (Anti-Echinococcus EUROLINE-WB®), using a panel of known status dog sera.

**Results:**

RecEm95-antigen was revealed to be the most promising antigen for use in ELISA, demonstrating 100% (95% CI: 72–100%) sensitivity and 100% (95% CI: 93–100%) specificity in our study. The in-house WB using EmVF antigen performed as well as the recEm95-ELISA. The commercial WB also correctly identified all infected dogs, coupled with a specificity of 98% (95% CI: 91–100%).

**Conclusion:**

The recEm95-ELISA alone or in combination with either the in-house WB or the Anti-Echinococcus EUROLINE-WB® (IgG) with a minor modification should be considered as the best current approach for the serological diagnosis of dogs infected with the larval stage of *E. multilocularis*. However, larger studies with a focus on potentially cross-reacting sera should be undertaken to verify these findings.

## Background

The etiological agent of alveolar echinococcosis (AE) is *Echinococcus multilocularis* in its larval (metacestode) form. Usually, the dog (in addition to the fox and other wild canids) acts as the definitive host in the life-cycle of the parasite. Relatively recently, domestic dogs have also been revealed to be aberrant intermediate hosts for AE [1]. Although not entailing large numbers of dogs, cases of canine AE have been regularly reported from Central Europe since 1988 and from Canada since 2009 (reviewed in [2]). It remains unclear why reports of canine AE are lacking from other highly endemic regions such as Central Asia, but possible explanations include underdiagnosis and reduced susceptibility of dogs to AE from local strains of the parasite. The most likely route of infection in dogs is ingestion of *E. multilocularis* eggs, either via contaminated food or water or coprophagia. Some authors have also postulated autoinfection with eggs from co-existing adult cestodes in the intestines [3, 4]. Unlike in humans [5], canine AE seems to develop rather quickly, as reflected by the young median age (3.1 and 4.1 years, respectively, in two unrelated studies) of affected dogs [6, 7]. Furthermore, AE in dogs is usually diagnosed only after the parasitic mass has grown to considerable size and abdominal dilatation is already present [6]; thus the disease has reached an advanced stage with severe health impairment. This could be responsible for the relatively widespread practice of euthanasia without attempting treatment for AE-affected dogs [6].

Diagnosis of canine AE typically follows initial detection of suspicious liver lesions on ultrasonographic examination. Etiological proof of diagnosis is then ascertained by polymerase chain reaction (PCR) or histology (periodic acid-Schiff staining) on fine needle aspirates or surgically resected tissues [6]. As these diagnostic approaches are invasive and most appropriate for rather advanced AE cases, the availability of non-invasive diagnostic techniques might help to yield earlier diagnosis of AE cases, which in turn favours successful treatment outcomes. Serology might thus be applied for routine screening of dogs with a high infection risk (i.e. those exhibiting coprophagia, hunting rodents or exposed to foxes and other definitive hosts), when suspicious hepatic structures are evidenced by imaging tools, or even for import screening of dogs travelling to *E. multilocularis*-free countries. Although most cases of canine AE are non-fertile as reported by Weiss et al. [8], Peregrine [9] has discussed the possible implications of importing a dog with AE to a non-endemic country. Serology for canine AE has been described by several authors (e.g. [4]), who used different antigens employed in enzyme-linked immunosorbent assays (ELISA). In a former study, we used an Em2-ELISA, in a protocol adapted from human medicine [6, 10]. For the serodiagnosis of human AE, the combination of various ELISAs and a Western blot (WB) is highly recommended as this combination has demonstrated a high diagnostic sensitivity and specificity [11]. The aim of this study was to search for the most suitable serological protocol for identifying metacestode-infected dogs with high sensitivity and specificity. Commercially available diagnostic kits and recombinant antigens were included as these could be widely applied.

## Methods

### Dog sera

#### Group 1: Known-infected dogs (clinical AE cases)

A total of 14 samples were obtained from confirmed cases of AE. Seven dogs had multiple liver lesions, as evidenced by abdominal ultrasound examination. One dog had a single lesion that measured about 15 cm in diameter. For the other 6 dogs, this information on the lesion (s) was unavailable. The disease was diagnosed either by PCR (13 dogs) or histology (1 dog). All dogs were privately owned. Serum samples were taken when the dogs were still alive, at the time of fine-needle aspiration of the liver lesions. Only one of the dogs was examined for concurrent intestinal infection with *E. multilocularis*, with a negative result on both flotation and copro-antigen ELISA.

#### Group 2: Presumed-uninfected dogs

A total of 41 samples were randomly selected from our serobank. Serum samples were obtained either from the small animal clinic from healthy blood donors or from submissions from local veterinarians for the serodiagnosis of other parasitic diseases, such as *Babesia canis*, *Leishmania infantum*, *Dirofilaria immitis* and *Neospora caninum*. No information on infection status with *E. multilocularis* was available for these dogs.

#### Group 3: Negative control group

A total of 20 samples were obtained from dogs housed in a controlled environment (experimental industrial dog facility at the Novartis Centre de Recherche in St-Aubin, Switzerland) throughout their life, receiving only a commercial food. Their health status was monitored on a daily basis and coprological examination for parasites was performed at least once per year, always with negative results. Serum samples were taken for general health observations and aliquots were sent to the Institute of Parasitology in Bern for this study. The dogs were kept according to Swiss animal welfare regulations.

### Parasite antigens and serological methods

Antigens used for ELISA were as follows: *E. multilocularis* vesicle fluid (EmVF) antigen was produced by in vitro cultivation and subsequent vesicle fluid collection as previously described [11]; Em2-antigen was purified by affinity chromatography as previously described [12]; recombinant Em18-antigen (recEm18) was produced according to Xiao et al. [13]; recombinant Em95 (recEm95) was synthesized according to the method described by Gauci et al. [14]. The antigen used for in-house WB was the same EmVF-antigen as that used for ELISA [11].

Coating of 96-well microplates for ELISA was done with 5 μg/ml EmVF- or with 0.5 μg/ml recombinant antigen (recEM95, recEM18) in coating buffer (bicarbonate/carbonate coating buffer (100 mM, pH 9.6) incubated overnight at 4 °C. Em2-antigen at 0.57 μg/ml coating buffer was used to coat wells according to Dai et al. [15]. After coating, plates were washed thrice with phosphate-buffered saline (PBS)-0.05%-Tween20, and then blocked with 100 μl per well sample diluent buffer (PBS-0.05%Tween20 with 0.05% bovine haemoglobin). Sera diluted 1:100 were added at 100 μl per well. Serum was incubated for 30 min at 37 °C. After three washings, a rabbit anti-dog Immunoglobulin G (IgG)-alkaline phosphatase conjugate (A0793, Sigma-Aldrich, Buchs, Switzerland) diluted 1:4000 in sample diluent buffer was added at 100 μl per well, subsequent incubation was for 30 min at 37 °C. After three washings as described above, 100 μl substrate solution (p-Nitrophenyl phosphate in 1 M diethanolamine, pH 9.8, with 0.5 mM MgCl_2_ buffer) was added and incubated for 15 min at 37 °C. The reaction was stopped with 100 μl of 3 M NaOH (100 μl/well). Plates were read at 405 nm with a Tecan sunrise ELISA reader using Magellan software (Tecan, Männedorf, Switzerland).

### Western blots

#### In-house WB

SDS-PAGE resolution of EmVF-antigen and electrotransfer onto nitrocellulose was performed as previously described by Müller et al. [11]. WB-strips were incubated with dog sera at a 1:10 dilution in PBS-Tween plus 5% skimmed-milk powder and then incubated overnight at 4 °C, gently agitated using a mechanical rocker. All subsequent steps were performed on the rocker. Four washes for 5 min each with PBS-Tween were then performed. This was followed by incubation with a rabbit anti-dog-IgG-horseradish-peroxidase conjugate (Sigma, A6792) at a dilution of 1:200 in PBS-Tween for 2 h at room temperature (RT). Washing of the nitrocellulose-strips was done with PBS-Tween (twice), followed by two washings with PBS only. DAB (one 3,3′-Diaminobenzidine tetrahydrochloride 10 mg tablet per 20 ml PBS buffer; Sigma D5905) was added as substrate with 10 μl of H_2_O_2_ and incubated for 15 min at RT. Afterwards, the strips were washed three times with distilled water to stop reactions. For banding pattern interpretation, sera were considered antibody-positive if any of the 8 kD or 21 kD bands were recognized by the serum. Of the total 75 samples available, only 72 dog sera were tested by in-house WB; no serum was left after ELISA testing for three dogs from group 2.

#### Anti-Echinococcus EUROLINE-WB® (IgG)

The Anti-Echinococcus EUROLINE-WB® (IgG) is a commercially available immunoblot system for the detection of antibodies against *Echinococcus* spp. in human sera. Antigens of *Echinococcus* sp., among others the p7 antigen, were electrophoretically separated and transferred to a membrane that was cut into thin strips. Additionally, three chips with recombinant antigens recEm18, recEm95, and recEgAgB were embedded onto each strip. Antibodies present in serum samples can thus bind to the whole parasite antigen as well as to the three defined antigens in the same assay. The test procedure was performed exactly as recommended by the manufacturer with the exception that, instead of an anti-human IgG conjugate, a rabbit anti-dog IgG-alkaline phosphatase conjugate (Sigma, A0793) diluted 1:200 was used. In brief, one strip per serum sample was blocked for 15 min with 1.5 ml of the universal buffer supplied with the kit. Sera were diluted 1:51 in the universal buffer (final volume 1.5 ml) and incubated at RT for 30 min while gently shaking. Then, strips were washed three times for 5 min with the universal buffer. Rabbit anti-dog IgG-alkaline phosphatase conjugate (Sigma, A0793) was diluted 1:200 in universal buffer and 1.5 ml were added per strip, followed by 30 min of incubation at RT, while shaking gently. Washing steps were repeated as above. Finally, 1.5 ml of the ready-to-use substrate solution contained in the kit was added to each strip, and the reactions allowed proceeding for 10 min. Strips were washed three times for 1 min with distilled water to stop reactions and subsequently analysed using the EUROLineScan software. Two sera of truly positive dogs could not be tested in the EUROLINE-WB® because there was not enough serum left.

### ROC analysis

The performance of each ELISA test was assessed by analysis of the receiver-operating characteristic (ROC) curve and the area under curve (AUC) [16]. This was done twice for each test, comparing results for dogs from group 1 (truly positive dogs) with those from dogs from groups 2 and 3 as well as comparing results for dogs from groups 1 and 3 only. The cut-off was chosen based on the point where the ROC curve was closest to a perfect discrimination between infected and non-infected dogs. Data analysis was performed with R software version 3.3.1 [17] with additional packages ROCR version 1.0–7 [18] and OptimalCutpoints version 1.1–3 [19].

## Results

**Fig. 1:**
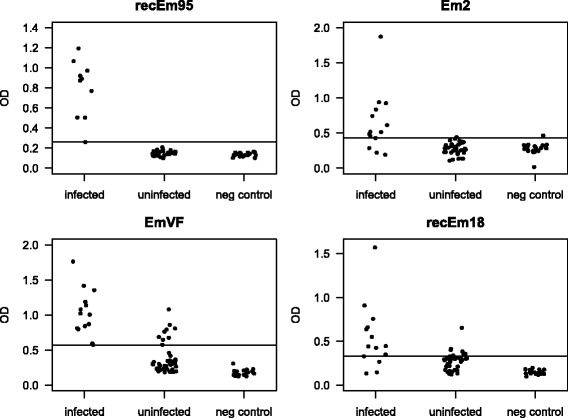
Results for all the sera in the different ELISAs. Cut-off values (horizontal lines) were established by ROC-analysis with all groups

The best performance on all tested sera (groups 1 to 3) was obtained with the recEm95 antigen: 100% (95% CI: 72–100%) sensitivity and 100% (95% CI: 93–100%) specificity (Fig. 1, Table 1). EmVF also had a very high sensitivity (100%; 95% CI: 78–100%), but a lower specificity of 85% (95% CI: 74–92%). Em2-antigen was able to distinguish between infected and uninfected dogs with a sensitivity of 79% (95% CI: 52–92%) and a specificity of 97% (95% CI: 89–99%). RecEm18 achieved the same sensitivity of 79% (95% CI: 52–92%), with the same specificity as EmVF (85%; 95% CI: 74–92%). When only groups 1 (truly positive dogs) and 3 (truly negative dogs) were compared, cut-off values were identical except that for recEm18 which was slightly lower (Table 2). Test performance in this comparison improved for recEm18 and even reached perfect sensitivity and specificity for EmVF. Performance remained unchanged for recEm95 and the specificity slightly decreased for Em2 (Table 2).

**Table 1:** Performance of each antigen in ELISA when including all three groups of dog sera. True positive sera (*n* = 14 of group 1) were analysed together with true negative sera (group 3) plus the selected sera from the serobank (group 2). This scenario yields a realistic impression of routine analysis of dogs for AE in Switzerland

ELISA	AUC	Cut-off	Sensitivity (95% CI)	Specificity (95% CI)
recEm95	1	0.261	1 (0.72–1)	1 (0.93–1)
Em2	0.837	0.430	0.79 (0.52–0.92)	0.97 (0.89–0.99)
EmVF	0.967	0.574	1 (0.78–1)	0.85 (0.74–0.92)
recEm18	0.838	0.329	0.79 (0.52–0.92)	0.85 (0.74–0.92)

*Abbreviations: AUC* area under curve, *CI* confidence interval

**Table 2:** Performance of each antigen in ELISA when including only known-infected vs known-uninfected dogs. True positive sera (*n* = 14 of group 1) were analysed against true negative sera (group 3) only. This scenario represents an ideal but unrealistic situation with a clear differentiation between positive and negative animals

ELISA	AUC	Cut-off	Sensitivity (95% CI)	Specificity (95% CI)
recEm95	1	0.261	1 (0.72–1)	1 (0.83–1)
Em2	0.821	0.430	0.79 (0.52–0.92)	0.95 (0.76–0.99)
EmVF	1	0.574	1 (0.78–1)	1 (0.84–1)
recEm18	0.932	0.268	0.86 (0.60–0.96)	1 (0.84–1)

*Abbreviations: AUC* area under curve, *CI* confidence interval

**Fig. 2:**
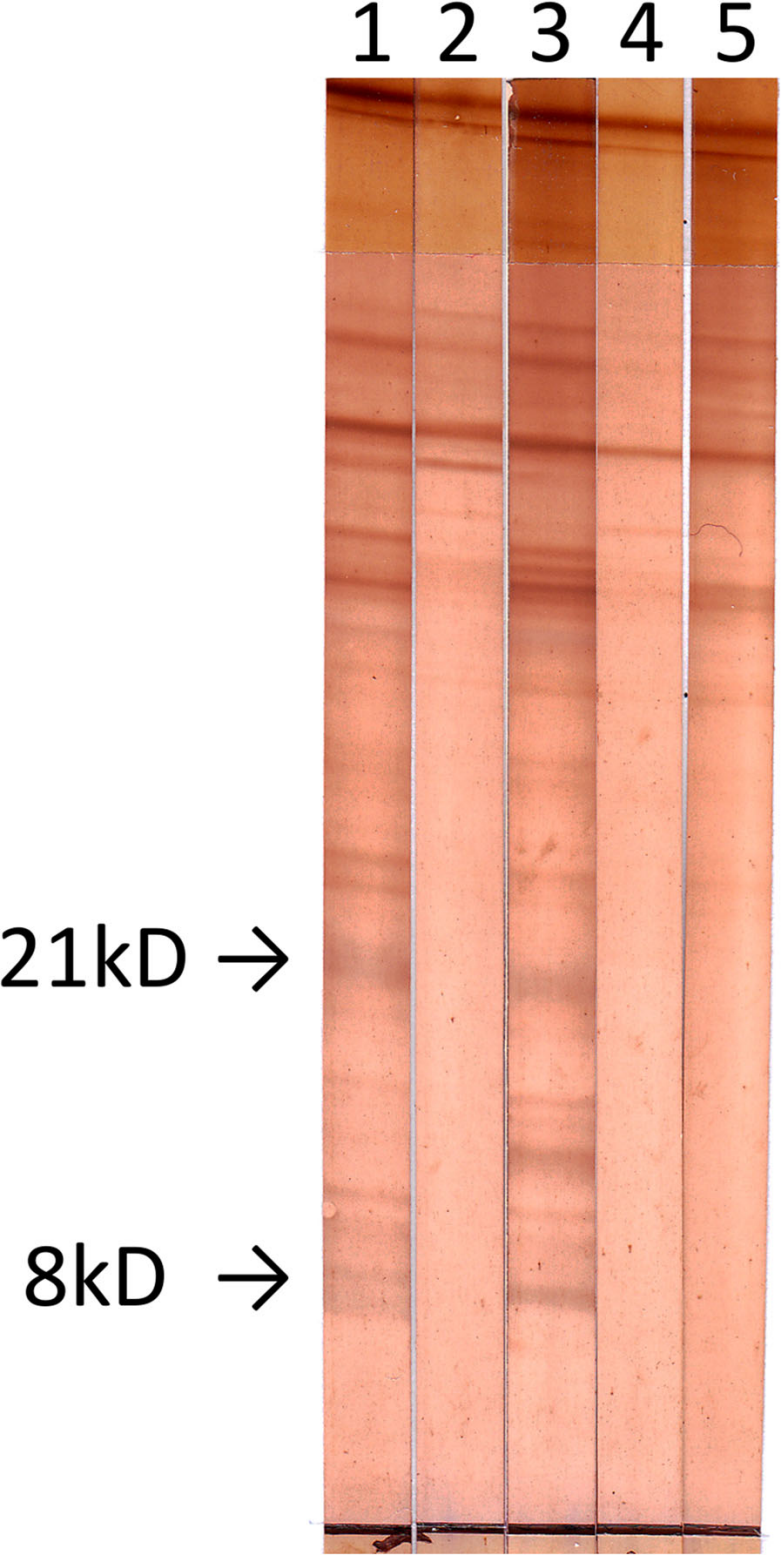
*E. multilocularis* Western-blot (in-house) with dog sera. Lanes 1, 3: infected dogs (group 1). Lane 2: known-uninfected dogs (group 3). Lanes 4, 5: presumably uninfected dogs (group 2)

Both WBs proved to be superior in both sensitivity and specificity to all ELISAs except the recEm95 ELISA. The in-house WB correctly identified all infected dogs as seropositive and all putatively uninfected and known uninfected dogs as seronegative [sensitivity = 100% (95% CI: 77–100%), specificity = 100% (95% CI: 94–100%)]. In detail, 10 sera recognized both bands (Fig. 2), 3 sera recognized the 21 kD band only, and a single serum sample reacted only with the 8 kD band (Table 3). As the WB was considered positive if any of the specific bands were recognized, all reactive sera were considered to be positive.

**Table 3:** Recognized bands in both WB per group of dog sera

Dog sera group	In-house^a^		EUROLINE-WB® (IgG)^a^	
	8 kD	21 kD	Em95	p7
Infected (group 1)	11/14	13/14	12/12	9/12
Uninfected (group 2)	0/38	0/38	1/41	0/41
Negative control (group 3)	0/20	0/20	0/20	0/20

^a^Indicated are: sera recognizing the band/all sera tested. When at least one of the bands was recognized by a serum, the result of the WB was interpreted as positive

**Fig. 3:**
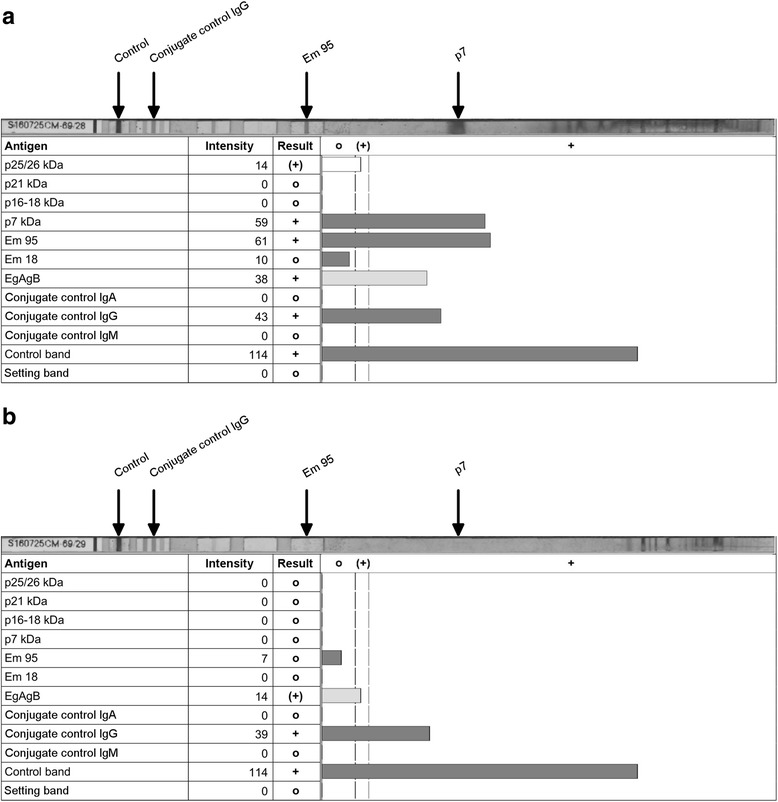
Anti-Echinococcus EUROLINE-WB® (IgG) with dog sera. **a** Serum of an infected dog (group 1). **b** Serum of an uninfected dog of group 2. The Anti-Echinococcus EUROLINE-WB® (IgG) is automatically read and interpreted by the EUROLineScan software. Horizontal bars represent intensity of the respective bands as automatically calculated by the software. Results are o = negative, (+) = borderline or + = positive

The modified Anti-Echinococcus EUROLINE-WB® (IgG) also recognized all positive sera as positive with 9 sera reacting with both the recEm95 and the p7 region, and 3 sera yielding a band only with the recEm95 antigen (Table 3, Fig. 3). In terms of specificity, one sample from group two (presumably negative dogs) weakly reacted with the Em95 region of the WB and was interpreted as positive by the analysing software. Sensitivity of the EUROLINE-WB® thus was 100% (95% CI: 74–100%) and specificity was 98% (95% CI: 91–100%).

## Discussion

Serodiagnosis of AE in dogs holds promise as an excellent tool to detect infected dogs at an early stage of infection and/or disease. Late diagnosis of disease often results in euthanasia or in partial surgical resection with a high risk of relapse [6]. Use of ELISA as a screening tool coupled with a confirmatory WB, as is the gold standard in human medicine [11], might also be an effective combination for dogs. Ideally, a recombinant antigen would be preferred for screening purposes, due to its amenability to large-scale production. In our study, the recombinant Em95 antigen proved to be very promising both for use in ELISA as well as in the EUROLINE-WB®. Also EmVF reached perfect sensitivity (95% CI: 78–100%) in our study, but specificity was poorer. The other antigens tested, which are used in screening ELISA’s for the detection of antibodies against *Echinococcus* spp. in human medicine, namely the native antigen Em2, as well as the recombinant Em18 yielded poorer results compared to those of recEm95. This was unexpected, since in most other intermediate host species infected with larval *E. multilocularis*, conventional antigens including Em2 and recEm18 have demonstrated the best diagnostic performance so far (reviewed in [20]). Furthermore, both WBs were superior to ELISA’s except for the recEm95 ELISA. As a stand-alone test or as a confirmatory test after screening with ELISA, the Anti-Echinococcus EUROLINE-WB® (IgG) has the advantage of being commercially available. However, the test system has been optimized for humans, and we have exchanged the conjugate in this study. The kit therefore might not yet be optimized for application in dogs, based on the one false positive and some borderline reactions encountered with the recEm95-antigen, which is blotted onto the WB strip. Unfortunately, the limited number of serum samples available did not enable further investigation. Reactions for sera from group 2 (presumed uninfected dogs) in the other ELISAs could have been due to infection with the intestinal stage of *E. multilocularis* [4] or to exposure to *E. multilocularis* eggs that resulted in a seroconversion against certain antigens, especially the Em2-antigen [21].

Unfortunately, we did not have access to known-status sera of dogs with intestinal *E. multilocularis* or other cestode infections. Sera of such animals were previously found to cross-react with most antigens used for serodiagnosis of canine AE [4] and should be included in future studies. Also, we did not have sera from dogs with other metacestode infections, such as *Mesocestoides* spp. or *Taenia crassiceps*. As well, sera from dogs with non-parasitic lesions that represent differential diagnoses for AE should be included in further studies to thoroughly validate the performance of the recEm95 ELISA as well as that of the two WBs.

The most exhaustive study to date to identify antigens for serodiagnosis of AE in dogs was carried out by Staebler et al. [4]. Antigens used by those authors included *E. multilocularis* Em2G11, EmII/3–10 (which diagnostically matches recEm18), protoscolex (EmP), excretory/secretory and adult integument (EmAd/I) antigens. Additionally, hydatid fluid (EgHF) and antigen B (EgAgB)) from *E. granulosus* were investigated [4]. The highest sensitivities for AE were obtained by the EmAd/I and the EmP antigens (97 and 93% sensitivity, respectively) with high specificities for the control group of 76 dogs (100 and 98.7%, respectively) [4]. Heterologous *E. granulosus* antigens (EgHF and EgAgB) showed low sensitivities (43 and 50%, respectively) with high numbers of false positive reactions (> 16%) in the control group [4]. However, the authors did not include WB in their study. Based on our experience, WB is the best performing method, although more laborious compared to ELISA. The most problematic serodiagnosis of AE we have experienced so far was in AE-affected beavers, whereby only WB was able to reliably diagnose infection [22]. Our finding that the recEm95-antigen, which was initially designed to vaccinate rodents against AE [14], also performs well for the detection of infection in an intermediate host species is new, and warrants further investigation of its use as a diagnostic antigen in other intermediate host species. RecEm95 is a homologue of Eg95, which is a dominant oncospheral antigen of *E. granulosus* [23]. In humans, recEm18 is the antigen of choice for follow-up of patients with AE [24], i.e. the serological response against this antigen correlates well with active and proliferating, as well as degenerating, metacestodes. It remains to be seen if there exists an antigen that will enable similar monitoring of the evolution of the metacestode infections in treated AE-dogs.

## Conclusion

Based on the results for the sera tested in this study, the recEm95-ELISA alone or in combination with either the in-house WB or the Anti-Echinococcus EUROLINE-WB® (IgG) with a minor modification should be considered the best current approach to the serological diagnosis of dogs infected with the larval stage of *E. multilocularis*. The advantage of recombinant antigens and commercially available tests are their broad availability and applicability to any diagnostic laboratory. However, more known status sera from dogs with intestinal *E. multilocularis* infections and from dogs suffering from other pathologies that constitute differential diagnoses for AE should be included in further studies to fully assess the performance of the most promising antigens identified in the present study.

## Abbreviations

AE: Alveolar echinococcosis
AUC: Area under curve
CI: Confidence interval
ELISA: Enzyme-linked immunosorbent assay
EmVF: *E. multilocularis* vesicle fluid
IgG: Immunoglobulin G
PBS: Phosphate-buffered saline
recEm18: Recombinant Em18
recEm95: Recombinant Em95
ROC: Receiver-operating characteristic
WB: Western blot
